# Proteinuria during Follow-Up Period and Long-Term Renal Survival of Childhood IgA Nephropathy

**DOI:** 10.1371/journal.pone.0150885

**Published:** 2016-03-15

**Authors:** Koichi Kamei, Ryoko Harada, Riku Hamada, Tomoyuki Sakai, Yuko Hamasaki, Hiroshi Hataya, Shuichi Ito, Kenji Ishikura, Masataka Honda

**Affiliations:** 1 Division of Nephrology and Rheumatology, National Center for Child Health and Development, Tokyo, Japan; 2 Department of Nephrology, Tokyo Metropolitan Children’s Medical Center, Fuchu, Tokyo, Japan; 3 Department of Pediatrics, Shiga University of Medical Science, Otsu, Shiga, Japan; 4 Department of Pediatric Nephrology, Toho University Faculty of Medicine, Tokyo, Japan; 5 Department of Pediatrics, Yokohama City University, Yokohama, Kanagawa, Japan; University of Glasgow, UNITED KINGDOM

## Abstract

**Background:**

Proteinuria is the most important risk factor for IgA nephropathy progression. The purpose of this study is to evaluate the long-term outcome and risk factors for poor prognosis in childhood IgA nephropathy.

**Methods:**

Patients who were diagnosed with IgA nephropathy between 1972 and 1992 at the Tokyo Metropolitan Kiyose Children’s Hospital were included. We analyzed risk factors for progression to end-stage kidney disease (ESKD) and chronic renal insufficiency (CRI) using Kaplan-Meier method and multivariate analyses of Cox proportional hazard model.

**Results:**

One hundred patients were included and the median observation period was 11.8 years. Twelve and 17 patients progressed to ESKD and CRI, respectively. The survival probabilities were 90.0% at 10 years and 79.8% at 20 years for ESKD, and 86.1% at 10 years and 72.3% at 20 years for CRI. Notably, patients with heavy proteinuria with hypoalbuminemia during follow-up period showed extremely poor prognosis. In this group, the survival rate at 10 years from ESKD and CRI was 40.6% and 20.8%, respectively. By multivariate analysis, proteinuria at diagnosis and proteinuria during follow-up period were risk factors for ESKD, whereas glomeruli showing mesangial proliferation ≥50% and proteinuria during follow-up period were risk factors for CRI. Patients without heavy proteinuria during follow-up period did not develop CRI and 63% of patients with mild proteinuria during follow-up period showed no proteinuria at the last observation.

**Conclusions:**

The degree of proteinuria during follow-up period is the strongest risk factor for ESKD and CRI.

## Introduction

IgA nephropathy is the leading cause of chronic glomerulonephritis worldwide today. Long-term follow-up studies revealed that 20–50% of adult patients progressed to end-stage kidney disease (ESKD) [1]. Heavy proteinuria, hypertension, decreased renal function at diagnosis have been identified as clinical risk factors while diffuse mesangial proliferation, crescents, segmental sclerosis, global sclerosis and interstitial fibrosis, and tubular atrophy have been reported as pathological risk factors for poor prognosis [1–7]. Among pediatric patients with IgA nephropathy in Japan, 11% have been reported to reach ESKD within 15 years [8], although there have been few reports regarding long-term prognosis of childhood IgA nephropathy. Risk factors for ESKD in childhood IgA nephropathy have also been reported [9–11]. As the incidence of pediatric IgA nephropathy patients who show hypertension or decreased renal function at onset is relatively rare in comparison with adults, proteinuria is the most important risk factor for progression of the disease in childhood.

Interestingly, there are reports that showed patients with heavy proteinuria or nephrotic syndrome at onset improved promptly with excellent outcome [12, 13]. On the other hand, in some cases with mild or no proteinuria at onset, the patients suffered from heavy proteinuria several years later and gradually progressed to chronic renal insufficiency (CRI) defined as estimated glomerular filtration rate (eGFR) < 60 mL/min/1.73m^2^ [14, 15]. Therefore, at onset of the disease it is difficult to predict the outcome. At present, there are several reports of proteinuria during follow-up period being a stronger predictor than proteinuria at onset in adult patients [16–19]. Recently, a report highlighted the relationship between proteinuria at two years after onset and the last observation in childhood IgA nephropathy [20]. However, there is no report regarding the relationship between proteinuria during the follow-up period and renal prognosis in childhood IgA nephropathy.

The purpose of this study is to examine the long-term outcome of patients with childhood IgA nephropathy and to evaluate the risk factors for ESKD and CRI, with a focus on proteinuria at onset and during follow-up period.

## Materials and Methods

### Study design and population

This is a retrospective observational study. One hundred thirteen patients were diagnosed with IgA nephropathy by renal biopsy between 1972 and 1992 at the Tokyo Metropolitan Kiyose Children’s Hospital (predecessor of Tokyo Metropolitan Children’s Medical Center). Among them, 13 patients were excluded as we could not follow medical records as they were treated by other institutes. So, data of 100 patients were available and they were included in this study. Although some of them have been transited to adult hospitals, we could get information of medical records about these 100 patients, including data of other hospitals. IgA nephropathy was diagnosed when mesangial hypercellularity with predominant IgA deposits was shown by pathological findings. Patients with Henoch-Schonlein purpura nephritis were excluded.

We obtained medical records containing information on gender, age at onset, age at diagnosis (first renal biopsy), initial presentation (screening, gross hematuria or edema), clinical parameters at diagnosis (serum creatinine level, serum albumin level, 24-h urinary protein excretion, blood pressure), pathological findings (rates of glomeruli showing mesangial proliferation, crescents and global sclerosis), treatment after diagnosis, degree of proteinuria during follow-up period and clinical features at the last observation.

We calculated eGFR at diagnosis using the Schwartz formula [21] for patients whose serum creatinine was measured by the Jaffe method before 1989. eGFR was calculated using enzymatic creatinine-based equation of Japanese children [22] for those whose serum creatinine was measured by an enzymatic method which was introduced in our hospital in 1989. Decreased renal function at diagnosis was defined as eGFR less than 90 mL/min/1.73m^2^. Serum albumin levels were measured by bromocresol green (BCG) method. Hypertension at diagnosis was defined as systolic or diastolic pressure that exceeded the 95^th^ percentile on the basis of age-matched standard values [23]. Urinary protein concentrations were measured by Pyrogallol Red Molybdate methods. Patients were grouped into four categories according to the degree of proteinuria defined by 24-h urinary protein excretion at diagnosis: group 1, no proteinuria (<0.2 g/day/1.73 m^2^); group 2, mild proteinuria (0.2–1.0 g/day/1.73 m^2^); group 3, heavy proteinuria without hypoalbuminemia (>1.0 g/day/1.73 m^2^ with serum albumin level ≥3.0 g/dL); and group 4, heavy proteinuria with hypoalbuminemia (>1.0 g/day/1.73 m^2^ with serum albumin level <3.0 g/dL). The degree of proteinuria during follow-up period was defined as the highest level of proteinuria during the period of more than one year after diagnosis and data which persisted at least more than six months were selected. We also categorized degrees of proteinuria during follow-up period into four groups (group 1–4). Urinary protein to creatinine ratio may be substituted by 24h-urinary protein excretion. For pathological findings, the rates of glomeruli (%) showing mesangial proliferation, crescents and global sclerosis were evaluated. Clinical features at the last observation were defined by five categories: no proteinuria (urinary protein to creatinine ratio <0.2) with normal renal function (eGFR ≥60 mL/min/1.73m^2^); mild proteinuria (0.2–1.0 of urinary protein to creatinine ratio) with normal renal function; heavy proteinuria (urinary protein to creatinine ratio >1.0) with normal renal function; CRI (eGFR < 60 mL/min/1.73m^2^) without ESKD; and ESKD (initiation of dialysis or transplantation). Observation period was defined as the time between diagnosis and the last visit. However, observation period of patients who developed ESKD was defined as the time until initiation of renal replacement therapy.

### Statistical analyses

The primary outcome was the progression to ESRD and CRI, which was analyzed with the Kaplan-Meier method. Risk factors for ESRD and CRI were calculated by univariate and multivariate analyses using Cox proportional hazard model. Clinical and pathological parameters (degree of proteinuria, renal function and presence of hypertension at diagnosis, rate of glomeruli showing mesangial proliferation, steroid treatment and degree of proteinuria during follow-up period) were selected for univariate analyses. Multivariate analyses were performed using statistically significant risk factors obtained by univariate analyses. Statistical significance was established at *P* <0.05. The data were analyzed with JMP version 11.0 (SAS Institute Japan Ltd., Tokyo, Japan).

### Ethics

The protocol of the present study was approved by the Ethics Committee of the Tokyo Metropolitan Children’s Medical Center (ID, H26-47). This study was conducted according to the Declaration of Helsinki. Records of patients were anonymized for personal information could not to be identified.

## Results

### Characteristics of patient

Characteristics of 100 patients are shown in Table 1. Their clinical features were stratified by degree of proteinuria at diagnosis. Twenty-five patients (25%) received steroid treatment which includes methylprednisolone pulse therapy or combination treatment (prednisolone, immunosuppressive agents, dipyridamole and warfarin) [24–26]. Steroid treatment was introduced two patients (6%) in group 2, 15 patients (33%) in group 3 and eight patients (42%) in group 4. Nonsteroidal agents include antiplatelet agents, anticoagulant agents and Sairei-to (Chinese-herb). No patients were treated with angiotensin-converting enzyme inhibitors, nor angiotensin receptor blockers as the initial treatment. Median follow-up period (period between diagnosis and last observation) was 11.8 years.

**Table 1:** Characteristics of patients.

	All patients (n = 100)	Degree of proteinuria at diagnosis			
		Group 1 (n = 3)	Group 2 (n = 32)	Group 3 (n = 46)	Group 4 (n = 19)
**Male gender**	**50 (50%)**	**3 (100%)**	**-44%**	**24 (52%)**	**9 (47%)**
**Age at onset (y)**	**10.1 ± 3.0**	**8.1 ± 1.9**	**10.4 ± 3.5**	**10.4 ± 2.8**	**9.4 ± 2.8**
**Age at diagnosis (y)**	**11.6 ± 3.2**	**9.3 ± 2.0**	**12.5 ± 3.7**	**11.7 ± 2.9**	**10.0 ± 2.7**
**Initial presentation**					
** Screening**	**70 (70%)**	**0 (0%)**	**20 (63%)**	**39 (85%)**	**11 (58%)**
** Gross hematuria**	**21 (21%)**	**3 (100%)**	**10 (31%)**	**6 (13%)**	**2 (11%)**
** Edema**	**9 (9%)**	**0 (0%)**	**2 (6%)**	**1 (2%)**	**6 (32%)**
**eGFR at diagnosis (ml/min/1.73 m**^**2**^**)**	**128.4 ± 35.7**	**148.3 ± 66.1**	**131.7 ± 36.5**	**128.3 ± 36.6**	**119.4 ± 26.0**
** ≧90**	**89 (89%)**	**3 (100%)**	**28 (88%)**	**40 (87%)**	**18 (95%)**
** 60–90**	**11 (11%)**	**0 (0%)**	**4 (13%)**	**6 (13%)**	**1 (5%)**
** 30–60**	**0 (0%)**	**0 (0%)**	**0 (0%)**	**0 (0%)**	**0 (0%)**
** 15–30**	**0 (0%)**	**0 (0%)**	**0 (0%)**	**0 (0%)**	**0 (0%)**
** <15**	**0 (0%)**	**0 (0%)**	**0 (0%)**	**0 (0%)**	**0 (0%)**
**Serum albumin level at diagnosis (g/dl)**	**3.7 ± 0.8**	**3.9 ± 0.5**	**4.3 ± 0.4**	**3.9 ± 0.4**	**2.3 ± 0.4**
**24-h urinary protein excretion at diagnosis (g/1.73 m**^**2**^**)**	**3.0 ± 2.9**	**0.1 ± 0.1**	**0.6 ± 0.2**	**2.9 ± 1.7**	**7.4 ± 2.3**
**Hypertension at diagnosis**	**20 (20%)**	**2 (67%)**	**6 (19%)**	**7 (15%)**	**5 (26%)**
**Pathological findings at diagnosis**					
** Glomeruli with mesangial proliferation (%)**	**51.3 ± 32.0**	**32.3 ± 30.4**	**30.1 ± 25.4**	**66.5 ± 29.3**	**55.2 ±27.6**
** Glomeruli with crescents (%)**	**4.3 ± 9.5**	**0.0 ± 0.0**	**2.8 ± 5.3**	**5.6 ±11.3**	**4.7 ±11.4**
** Glomeruli with global sclerosis (%)**	**4.0 ± 9.3**	**0.0 ± 0.0**	**1.9 ± 6.2**	**3.8 ± 5.8**	**10.4 ±18.4**
**Treatment**					
** Steroid**	**25 (25%)**	**0 (0%)**	**2 (6%)**	**15 (33%)**	**8 (42%)**
** Nonsteroidal agents**	**59 (59%)**	**3 (100%)**	**20 (63%)**	**26 (57%)**	**10 (53%)**
** No treatment**	**16 (16%)**	**0 (0%)**	**10 (31%)**	**5 (11%)**	**1 (5%)**
**Observation period (y)**	**11.8 (0.8–24.8)**	**16.7 (3.6–16.7)**	**12.8 (4.9–24.8)**	**7.0 (2.2–19.8)**	**10.6 (0.8–19.4)**
**Clinical features at the last observation**					
** No proteinuria with normal renal function**	**52 (52%)**	**3 (100%)**	**22 (69%)**	**20 (43%)**	**7 (37%)**
** Mild proteinuria with normal renal function**	**21 (21%)**	**0 (0%)**	**9 (28%)**	**11 (24%)**	**1 (5%)**
** Heavy proteinuria with normal renal function**	**10 (10%)**	**0 (0%)**	**0 (0%)**	**8 (17%)**	**2 (11%)**
** Chronic renal insufficiency**	**5 (5%)**	**0 (0%)**	**0 (0%)**	**4 (9%)**	**1 (5%)**
** End-stage kidney disease**	**12 (12%)**	**0 (0%)**	**1 (3%)**	**3 (7%)**	**8 (42%)**

Data are expressed as means±SD or median (min-max) or number of patients (%).

**Fig 1:**
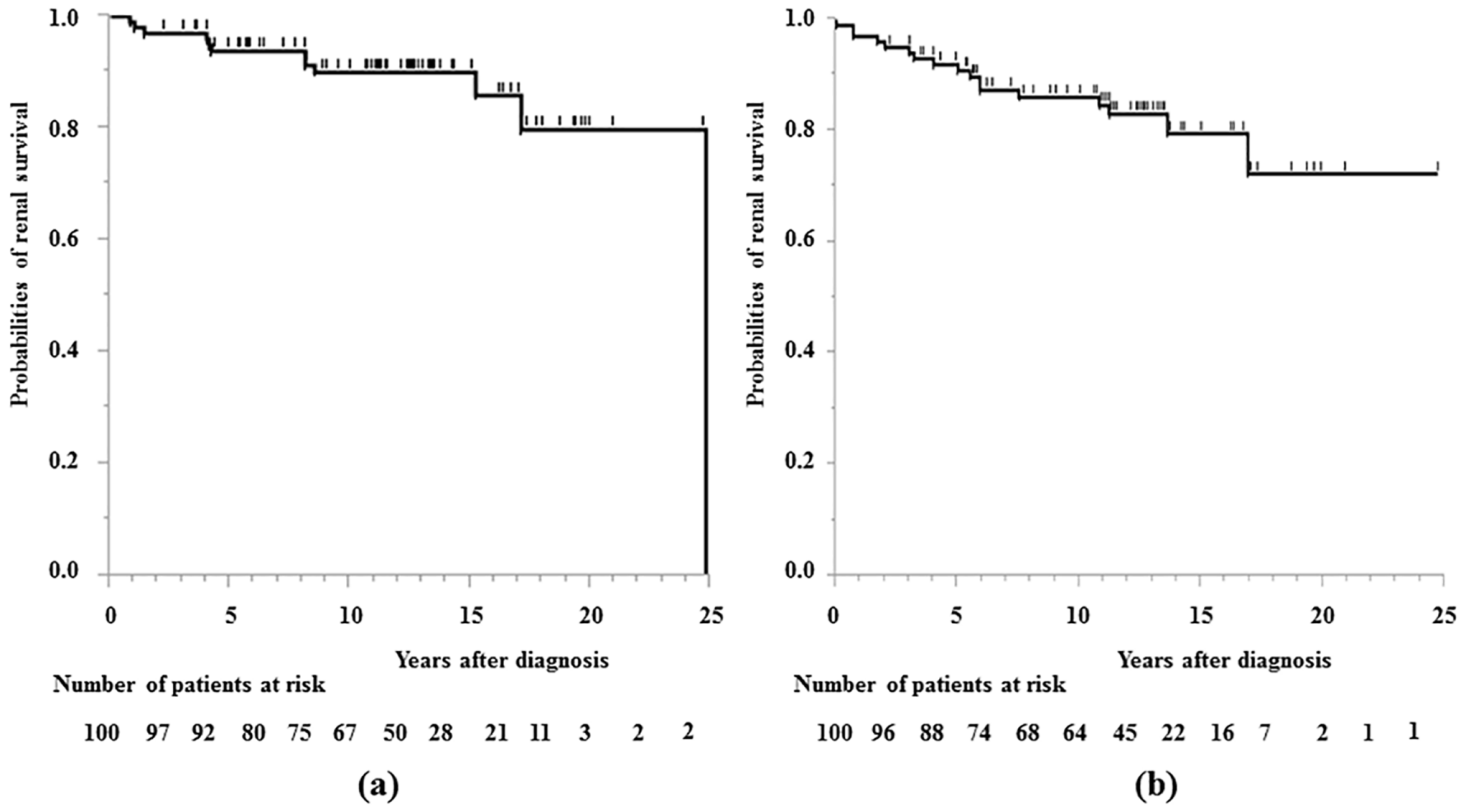
Renal survival curve of all patients. a) Kaplan-Meier plot showing time to ESKD. b) Kaplan-Meier plot showing time to CR.

Fig 1 shows the Kaplan-Meier analysis for the time to ESKD and CRI. Twelve patients (12%) reached ESKD and the median duration until ESKD was 6.2 years (0.8–24.8 years). The renal survival probabilities were 93.8% at 5 years, 90.0% at 10 years, 90.0% at 15 years and 79.8% at 20 years. Seventeen patients (17%) reached CRI and the survival probabilities were 91.9% at 5 years, 86.1% at 10 years, 79.5% at 15 years and 72.3% at 20 years.

### Renal survival stratified by proteinuria

**Fig 2:**
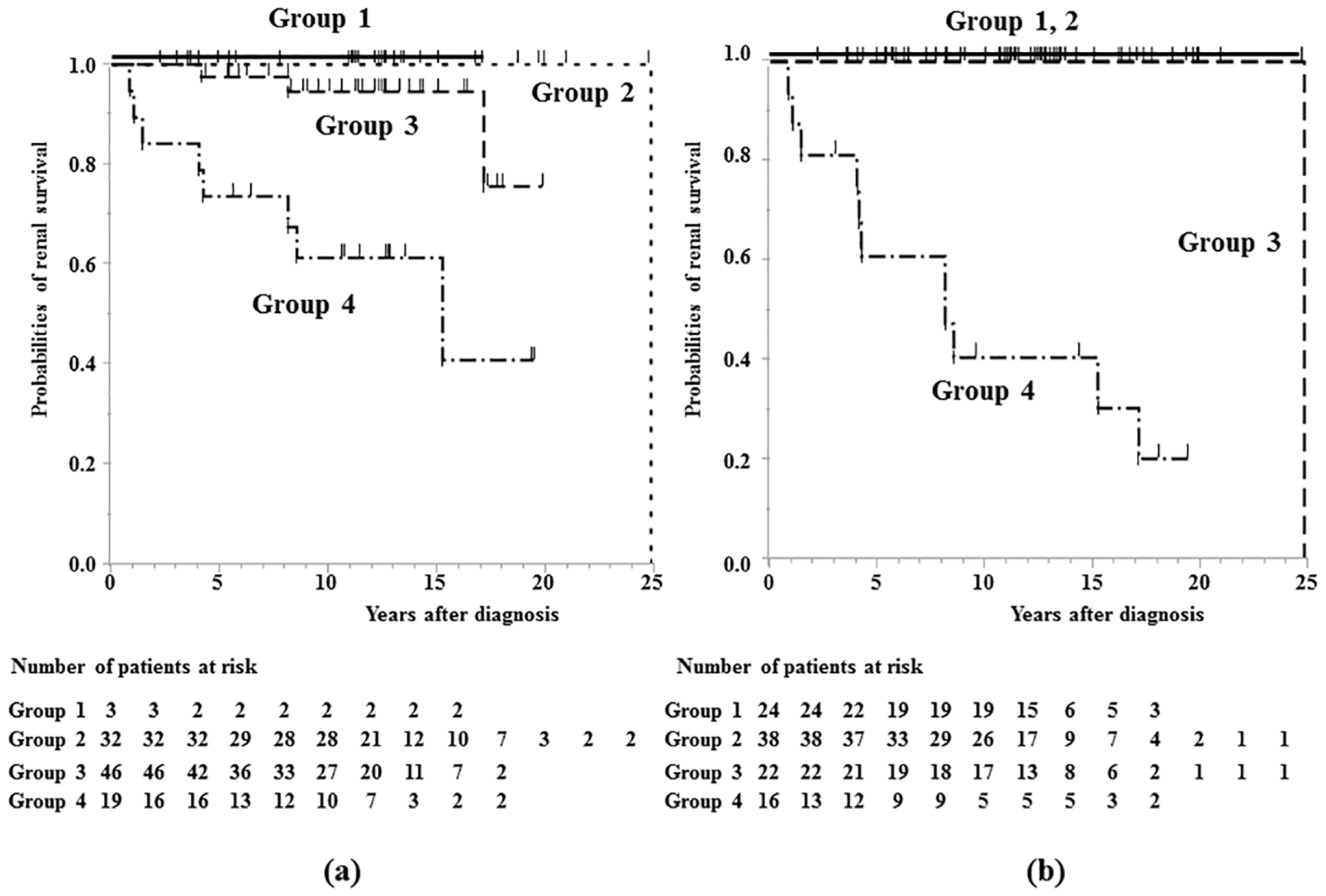
Survival curve showing time to ESKD. a) Kaplan-Meier plot showing time to ESKD stratified by proteinuria at diagnosis. b) Kaplan-Meier plot showing time to ESKD stratified by proteinuria during follow-up period. group 1, no proteinuria (<0.2 g/day/1.73 m^2^); group 2, mild proteinuria (0.2–1.0 g/day/1.73 m^2^); group 3, heavy proteinuria without hypoalbuminemia (>1.0 g/day/1.73 m^2^ with serum albumin level ≥3.0 g/dL); group 4, heavy proteinuria with hypoalbuminemia (>1.0 g/day/1.73 m^2^ with serum albumin level <3.0 g/dL).

**Fig 3:**
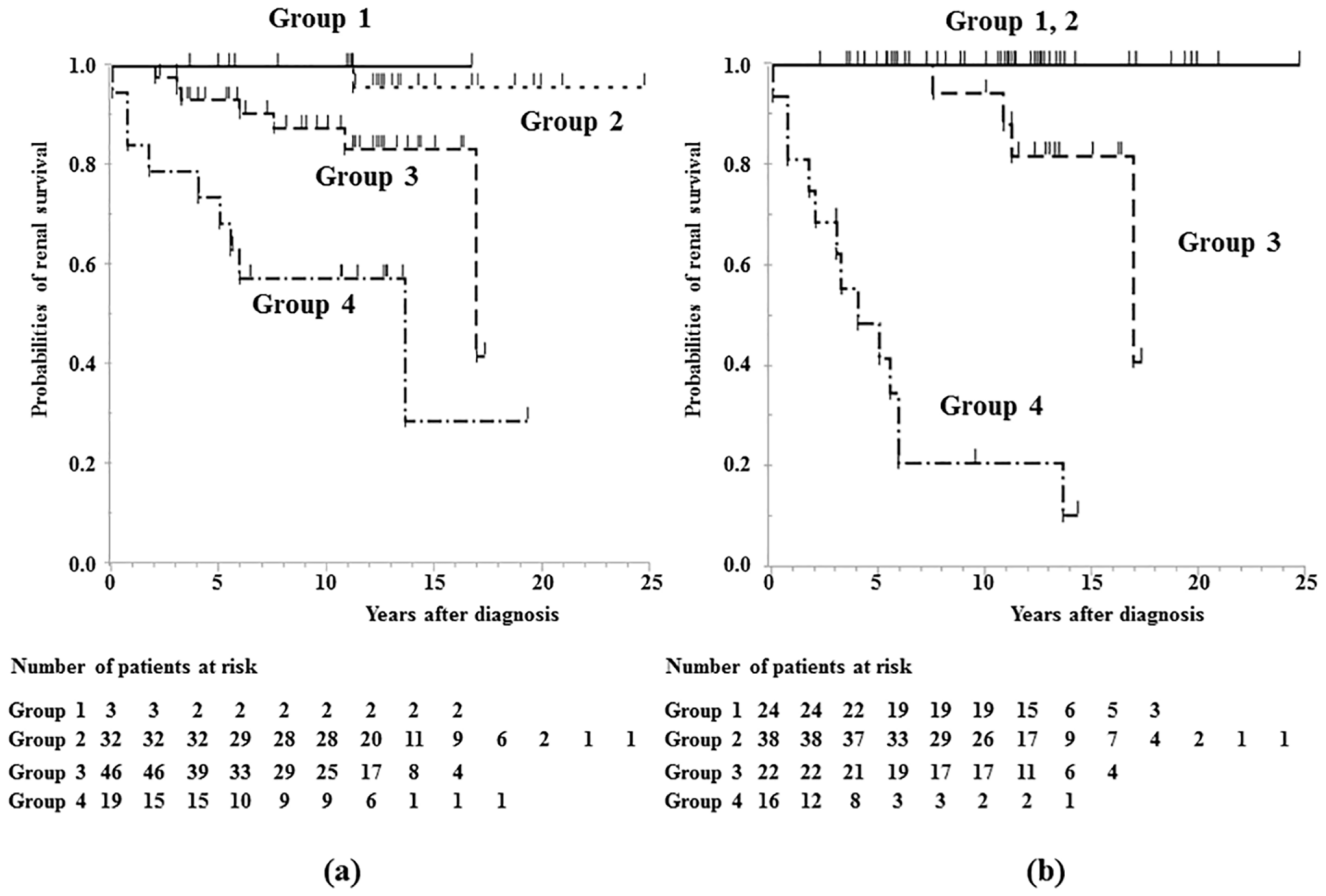
Survival curve showing time to CRI. a) Kaplan-Meier plot showing time to CRI stratified by proteinuria at diagnosis. b) Kaplan-Meier plot showing time to CRI stratified by proteinuria during follow-up period. group 1, no proteinuria (<0.2 g/day/1.73 m^2^); group 2, mild proteinuria (0.2–1.0 g/day/1.73 m^2^); group 3, heavy proteinuria without hypoalbuminemia (>1.0 g/day/1.73 m^2^ with serum albumin level ≥3.0 g/dL); group 4, heavy proteinuria with hypoalbuminemia (>1.0 g/day/1.73 m^2^ with serum albumin level <3.0 g/dL).

We compared the prognosis (ESKD and CRI) using Kaplan-Meier analysis stratified by proteinuria at onset and during follow-up period. The survival rates from ESKD were 73.7% at 5 years, 61.4% at 10 years and 61.4% at 15 years in group 4 patients at diagnosis, while they were 60.9% at 5 years, 40.6% at 10 years and 40.6% at 15 years in group 4 patients during follow-up period (Fig 2). The survival rates from CRI were 68.4% at 5 years, 57.4% at 10 years and 28.7% at 15 years in group 4 patients at diagnosis, while they were 41.7% at 5 years, 20.8% at 10.0 years in group 4 patients during follow-up period (Fig 3). No patients in groups 1 and 2 during follow-up period developed CRI.

**Fig 4:**
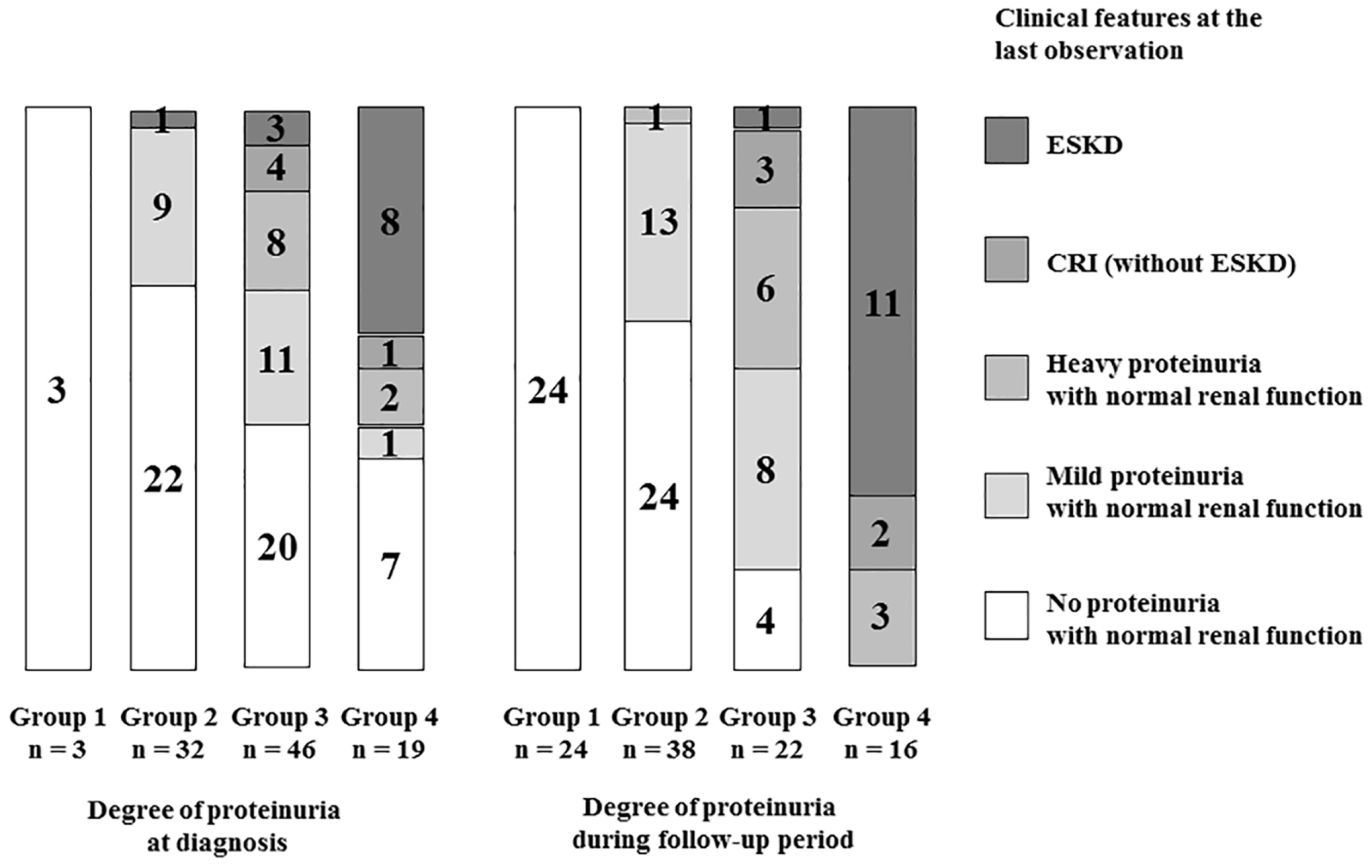
Relationship between degree of proteinuria (at diagnosis/during follow-up period) and clinical features at the last observation. Group 1, no proteinuria (<0.2 g/day/1.73 m^2^); group 2, mild proteinuria (0.2–1.0 g/day/1.73 m^2^); group 3, heavy proteinuria without hypoalbuminemia (>1.0 g/day/1.73 m^2^ with serum albumin level ≥3.0 g/dL); group 4, heavy proteinuria with hypoalbuminemia (>1.0 g/day/1.73 m^2^ with serum albumin level <3.0 g/dL).

**Fig 5:**
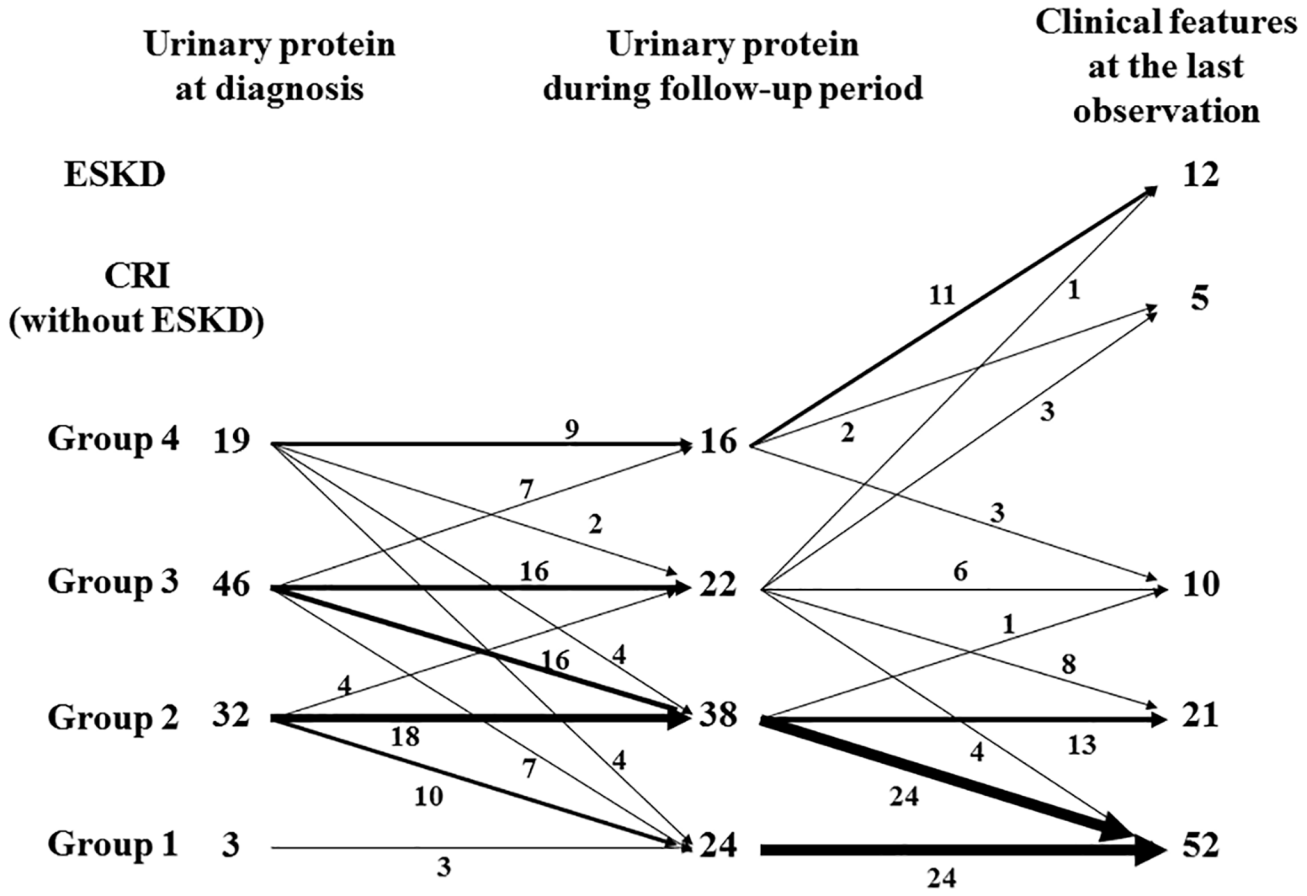
Clinical course of all patients. Group 1, no proteinuria (<0.2 g/day/1.73 m^2^); group 2, mild proteinuria (0.2–1.0 g/day/1.73 m^2^); group 3, heavy proteinuria without hypoalbuminemia (>1.0 g/day/1.73 m^2^ with serum albumin level ≥3.0 g/dL); group 4, heavy proteinuria with hypoalbuminemia (>1.0 g/day/1.73 m^2^ with serum albumin level <3.0 g/dL).

Fig 4 shows the relationship between the degree of proteinuria (at diagnosis/during follow-up period) and clinical features at the last observation. Eleven of 16 patients (69%) of group 4 during follow-up period developed ESKD at the last observation, while 8 of 19 patients (42%) of group 4 at diagnosis developed ESKD. Almost half patients of group 4 at diagnosis showed no or mild proteinuria at the last observation, while none of group 4 during follow-up period showed such beneficial outcome at the last observation. One patient with mild proteinuria at diagnosis showed heavy proteinuria during follow-up period and developed ESKD at the last observation. Fig 5 shows the clinical courses of all patients. All patients with poor prognosis (ESKD or CRI) were in group 3 or 4 during follow-up period, whereas no patients in group 1 or 2 during the same period had poor prognosis. Twenty-four of 38 patients (63%) with mild proteinuria (group 2) during follow-up period showed no proteinuria at the last observation.

### Risk factors for ESKD and CRI

Tables 2 and 3 shows the factors associated with progression to ESKD and CRI. By univariate analysis, group 4 at diagnosis, glomeruli showing mesangial proliferation ≥50% and group 4 during follow-up period were calculated as risk factors for ESKD. In these factors, group 4 at diagnosis and group 4 during follow-up period were calculated as statistically significant risk factors for ESKD by multivariate analysis (Table 2). As for risk factors for CRI, group 4 at diagnosis, glomeruli showing mesangial proliferation ≥50%, steroid treatment and group 4 during follow-up period were calculated as risk factors by univariate analysis. In these factors, glomeruli showing mesangial proliferation ≥50% and group 4 during follow-up period were calculated as statistically significant risk factors for CRI by multivariate analysis (Table 3).

**Table 2:** Predicted risk factors for end-stage kidney disease.

	Univariate		Multivariate	
	HR (95%CI)	P value	HR (95%CI)	P value
**Group 4 at diagnosis**	**13.6 (3.9–62.4)**	**< 0.0001**	**5.4 (1.1–44.8)**	**0.04**
**eGFR < 90 ml/min/1.73 m2 at diagnosis**	**0.8 (0.04–4.0)**	**0.79**		
**Hypertension at diagnosis**	**0.4 (0.02–2.1)**	**0.32**		
**Glomeruli with mesangial proliferation ≧50% at diagnosis**	**8.9 (1.6–165.8)**	**0.008**	**1.4 (0.1–30.1)**	**0.79**
**Steroid treatment**	**2.6 (0.7–8.7)**	**0.13**		
**Group 4 during follow-up period**	**(**a**)**	**< 0.0001**	**(**a**)**	**< 0.0001**

HR, hazard ratio; 95%CI, 95% confidence interval

^a^Hazard ratio cannot be obtained since all events were in group 4 during follow-up period.

**Table 3:** Predicted risk factors for chronic renal insufficiency.

	Univariate		Multivariate	
	HR (95%CI)	P value	HR (95%CI)	P value
**Group 4 at diagnosis**	**6.5 (2.5–17.6)**	**0.0002**	**2.4 (0.6–9.5)**	**0.2**
**eGFR < 90 ml/min/1.73 m2 at diagnosis**	**2.1 (0.5–6.4)**	**0.29**		
**Hypertension at diagnosis**	**0.5 (0.08–1.8)**	**0.32**		
**Glomeruli with mesangial proliferation ≧50% at diagnosis**	**13.7 (2.7–249.8)**	**0.0004**	**8.1 (1.3–165.8)**	**0.03**
**Steroid treatment**	**4.6 (1.7–13.1)**	**0.003**	**0.6 (0.1–2.3)**	**0.43**
**Group 4 during follow-up period**	**49.9 (15.5–222.1)**	**< 0.0001**	**78.0 (13.8–730.5)**	**< 0.0001**

HR, hazard ratio; 95%CI, 95% confidence interval

## Discussion

To date, few reports on the long-term prognosis of patients with childhood-onset IgA nephropathy are available [8, 27, 28]. The median observation period of our study was 11.8 years, which is much longer than previous reports. Two-thirds of all patients were followed for more than 10 years. Moreover, several patients who developed ESKD completed the observation period within 10 years due to the initiation of renal replacement therapy. Our study is also the first report on the relationship between proteinuria during the follow-up period and renal prognosis in childhood IgA nephropathy.

Han et al. reported that five of 24 adult patients (21%) with nephrotic onset IgA nephropathy underwent spontaneous remission within six months after onset [12]. Kim et al. summarized 100 adult patients with nephrotic onset IgA nephropathy and reported that complete remission, partial remission and no response occurred in 48%, 32% and 20% of patients, respectively, while 24% of them underwent spontaneous remission [13]. In our experience of proteinuria with hypoalbuminemia at diagnosis, 37% showed no proteinuria and 42% developed ESKD at the last observation, which is similar to previous reports. We speculate that some cases of IgA nephropathy of nephrotic onset have a self-limiting character, similar to acute glomerulonephritis and purpura nephritis. On the other hand, nephrotic syndrome or heavy proteinuria during follow-up period may indicate failure of treatment, thus explaining the poor prognosis.

There are also reports of disease activity worsening after several years in patients who showed no or slight proteinuria at diagnosis. Szeto et al. reported that 33% of 72 adult patients with mild proteinuria (0.4 g/day or less) at onset developed proteinuria of 1 g/day or more during a median follow-up of seven years [14]. Shen et al. reported that 29% of 135 adult patients with isolated microscopic hematuria of IgA nephropathy developed proteinuria, and 20% developed renal insufficiency during a median follow-up of 92 months [15]. In view of these reports, one cannot be optimistic about the prognosis even if proteinuria is mild at onset.

In this study we proved that the degree of proteinuria during follow-up period strongly correlated with the final prognosis. Although a certain degree of proteinuria at onset is a significant risk factor, it is not an absolute factor for prognosis. It remains difficult to predict the outcome of each patient based on the clinical and pathological parameters at onset. A similar result has been reported in adult patients [16–19]. In particular, heavy proteinuria with hypoalbuminemia during follow-up period indicated a poor prognosis, with two-thirds of those patients developed ESKD, and others developed CRI or heavy proteinuria at the last observation. None of them showed no or mild proteinuria at the last observation. On the other hand, even if patients showed heavy proteinuria during follow-up period, they are less prone to develop ESKD within a few years without hypoalbuminemia, based on our observations. Patients with mild proteinuria during follow-up period showed excellent prognosis and none of them showed CRI. Moreover, two-thirds of them showed no proteinuria at the last observation. For patients with mild proteinuria, we propose that strong immunosuppression should be avoided. As a goal of the treatment of IgA nephropathy, we should aim to reduce 24-h proteinuria to less than 1 g/1.73 m^2^ (or 1 g/g of urinary protein to creatinine ratio) to reduce the possibility of progression to CRI.

There are several limitations to our study. First, we selected the highest level of proteinuria, not time-average proteinuria [16, 17], in the follow-up period. It was difficult to calculate time-average proteinuria as data of urinary protein concentrations were often lacking, and only dipstick tests were performed in some patients who showed no or slight proteinuria. Second, all of the pathological findings were obtained at diagnosis, not at the period when the disease activity became high during the follow-up period. Third, as serum creatinine measurements were different before and after 1989, there might be slight errors in calculated data of eGFR. Forth, the sample size was relatively small. For validation of our results, a larger sample cohort is required. Finally, as the period of diagnosis was in the relatively distant past, indication of treatment, such as steroid, is not clear.

In conclusion, the degree of proteinuria during follow-up period is the strongest risk factor for IgA nephropathy progression in children. In particular, proteinuria during follow-up period shows absolutely poor prognosis. Patients who have no heavy proteinuria during follow-up period hardly develop renal insufficiency. Pediatric nephrologists should plan the treatment strategy with the long-term outcome in mind.
